# Assessment of Manual Abilities Using the Box and Block Test in Children with Bilateral Cerebral Palsy

**DOI:** 10.1155/2022/9980523

**Published:** 2022-02-22

**Authors:** Vanessa Zapata-Figueroa, Fernando Ortiz-Corredor

**Affiliations:** ^1^Department of Physical Medicine and Rehabilitation, Universidad Nacional de Colombia, Carrera 45 # 26-85, Bogotá D.C. 111321, Colombia; ^2^Centro de Investigación en Fisiatría y Electrodiagnóstico (CIFEL), Calle 26 # 69c-03, Bogotá D.C. 110931, Colombia; ^3^Department of Physical Medicine and Rehabilitation, Instituto Roosevelt, Carrera 4 Este # 17-50, Bogotá D.C. 111711, Colombia; ^4^Universidad Nacional de Colombia, Carrera 45 # 26-85, Bogotá D.C. 111321, Colombia

## Abstract

**Objective:**

The aim of this study was to determine the correlation between manual dexterity evaluated with the Box and Block Test (BBT) and the performance of daily activities in children with bilateral cerebral palsy (CP).

**Methods:**

The BBT was applied to 162 children with cerebral palsy of bilateral distribution aged 6 to 13 years. The level of performance was evaluated according to the Manual Ability Classification System (MACS), Gross Motor Function Classification System (GMFCS), and Pediatric Evaluation of Disability Inventory (PEDI) in the domains of self-care, mobility, and social function. Correlations between the findings of the BBT and the PEDI were determined, and additionally, some specific toileting tasks of the PEDI were evaluated.

**Results:**

The results of the BBT were lower in the lowest functional levels of the MACS (*p* ≤ 0.001). The BBT showed a strong correlation with the domains' self-care (*r* = 0.8), mobility (*r* = 0.7), and social function (*r* = 0.6) of the PEDI. The BBT was different between children who were able and children who were not able to perform the toileting tasks that were evaluated. A lower capacity in the BBT obtained in children with functional status GMFCS III, IV, and V was associated with poor performance in toileting tasks evaluated in the PEDI.

**Conclusion:**

The results of the BBT are correlated with the activities of daily living of children with bilateral CP. The data obtained from this test is used to predict the performance of daily activities of these patients in settings such as school and home and helps to identify contextual factors that influence the level of independence in children with bilateral CP.

## 1. Introduction

The functional independence of children with cerebral palsy (CP) is determined by multiple factors, such as cognitive ability, gross motor function, and manual dexterity. Manual dexterity involves complex abilities that allow for rapid and coordinated hand movements and that require proper integration of the upper limb with the central nervous system. Gross manual dexterity is strongly related to manual abilities required for a child's functional independence [1].

Manual ability in children with bilateral CP is affected to different degrees, compromising their independence. Therefore, therapeutic activities for the upper limb are an important part of the rehabilitation program and serve to define treatment goals. On the other hand, therapeutic goals are defined based on a consensus between health professionals, patients, and their families. Goals are established according to the family's priorities, children's performance in activities of daily living, and their abilities in standardized settings.

Children's performance in the domains of self-care, mobility, and social function is evaluated through questionnaires applied to the family. Different assessment scales that apply in clinical settings serve to predict achievements in children's performance in activities of daily living. Some level of consistency and correlation between family information and tests applied directly in clinical settings should be expected. On some occasions, families may underestimate or overestimate the child's actual performance. The activities that children perform or do not perform do not always coincide with the evaluation of therapists. Consequently, it is necessary to standardize and categorize the evaluations carried out, following the model of the International Classification of Functioning, Disability, and Health (ICF).

The ICF is a classification of health and health-related conditions designed for children and adults. The ICF framework consists of two parts: functioning and disability (including changes in body function and structure and assessment of capacity and performance) and contextual factors (including environment and personal factors).

Capacity describes a person's ability to execute a task and is explored by applying tests in controlled and standardized environments. Performance describes what children do in current environments. Having both assessments contribute to set treatment goals, but it is important that there is some degree of correlation between the capacity test used and the child's performance in daily life.

The analysis of the relationship between capacity and performance helps to identify the presence of contextual factors, which interfere in the performance of activities of daily living. In other words, if capacity is less than performance, it is probably that children's current environment has enabled them to perform better than what data about capacity would predict. However, if capacity is greater than performance, some aspects of the child's environment should be considered barriers to performance (physical barriers, culture, and attitudes of other people towards the child with disabilities), and they must be identified to guide the therapeutic goals.

The PEDI is used to describe children's functional status in the domains of self-care, mobility, and social function [2]. It has been used to measure performance and capacity in children with CP [3]. The functional level of the upper limbs according to the PEDI is correlated with the results of the MACS [4].

Child development involves demanding daily tasks such as activities related to hygiene and toileting skills. Children with CP may need physical environment modifications, especially to use toilet facilities at home, school, and community. These adaptations are priorities mentioned in different studies [5].

Children are expected to improve their independence in performing toileting skills as their developmental process progresses that can be assessed with several items of the PEDI. For example, the skill of wiping themselves thoroughly after bowel movement (item 63 of self-care domain) is the most complex toileting skill required for functional independence of that domain and the third most difficult task of the 73 items in the self-care domain [2, 6]. By 4 years of age, half of the children are able to wipe effectively by themselves [6]. After 6 years of age, more than 90% of normally developing children are able to perform this activity [2].

A high percentage of children in functional status IV and V do not have adequate sphincter control [7]. Many children with CP require full assistance at home, school, and the community for these types of activities. Educational barriers are often determined by children's toileting difficulties. Many children with bilateral CP are attending school with a caregiver to help them with these tasks.

Various scales and clinical tools have been proposed to assess upper limb capacity in children with CP (evaluation in a controlled environment) [8]. In the context of the IFC model, skills such as grasping, lifting, manipulating, reaching, turning, and releasing objects are found in the component of activities and can be explored in a controlled environment with various assessment tools. One such tool is the Box and Block Test.

The Box and Block Test (BBT) assesses unilateral gross manual dexterity. This tool is a quick and practical test that has been used to assess children with CP [9, 10]. For instance, it has been used to measure therapeutic outcomes and validate clinical assessment tools in CP [11–13]. A correlation between the BBT and other manual ability tests has been shown in healthy children and children with CP [14, 15]. Furthermore, a strong correlation has been found between the BBT and activities of daily living [16–19].

One of the advantages of the BBT is the ease of application. Furthermore, added to the assessment of grasping and manual dexterity, it involves visual-motor coordination, a function that can also be affected in children with CP. The BBT has shown a significant correlation with the performance of children with CP evaluated with the PEDI [18].

The main objective of our study was to determine the relationship between the results of the BBT and performance in each of the three domains (self-care, mobility, and social function) of the PEDI in children with bilateral CP. A secondary objective was to establish the relationship of the BBT with performance of specific toileting tasks evaluated in the PEDI.

## 2. Materials and Methods

### 2.1. Patients

All children seen at the Instituto Roosevelt between May 2013 and June 2017 with a diagnosis of bilateral CP were included. In our institute, all children with CP are systematically assessed using questionnaires and standardized clinical tests. In all cases, an electronic record of data is kept using FileMaker Pro® software (FileMaker Inc., Santa Clara, CA, USA).

Only children with CP of bilateral distribution were included, aged 6 years or older and under 13 years of age, who were able to pass at least one block in the BBT with either hands. Children who could not perform the test, due to cognitive limitations to follow the instruction, were excluded.

### 2.2. Box and Block Test

The Box and Block Test is used to assess gross manual dexterity. The BBT was applied according to the specifications previously described [20]. A 15-second trial period was performed. The test always started with the right hand; the height of the chair was controlled in order for all children to be able to put their feet on the floor; the height of the elbows always remained at the level of the table. For the analysis, the average of the two sides was calculated.

### 2.3. GMFCS

The Gross Motor Function Classification System (GMFCS E&R) is composed of 5 levels. Distinctions between one level and another are based on functional limitations and the need for handheld mobility devices (crutches, canes, and walkers) or wheeled mobility. The Spanish version of GMFCS was used in the study (available at https://canchild.ca/en/resources/42-gross-motor-function-classification-system-expanded-revised-gmfcs-e-r.

### 2.4. MACS

The Manual Ability Classification Scale (MACS) was applied in all cases [21]. The MACS scale helps to describe how children use their upper limbs to handle objects in daily activities and facilitates communication in the clinical setting. Each level is determined based on the child's usual activities and the degree of assistance required: Level I handles objects easily and successfully; level II handles most items, but with reduced quality and/or speed of achievement; level III handles objects with difficulty and needs help to prepare and/or modify activities; level IV handles a limited selection of easily managed objects in adapted situations; and level V does not handle objects and has severely limited ability to perform even simple actions. The MACS classification was used according to the Spanish version published by the authors of the scale. It is available at http://www.macs.nu/download-content.php.

### 2.5. PEDI

The PEDI questionnaire assesses what the child does in a daily environment. The questionnaire has been used in multiple disabling diseases in children, including CP [22].

It is a tool that evaluates different activities in the domains of self-care, mobility, and social function [2]. It is applied in children from 6 months to 7.5 years old or in older children with developmental delay. There are 73 items in the self-care domain, 59 items in the mobility domain, and 65 items in the social function domain [2].

The PEDI questionnaire was applied according to the manual using a validated version in Spanish [23]. For the statistical analysis, scaled scores (0-100) obtained from the raw results were used.

### 2.6. Statistical Analysis

For the presentation of continuous data (age, PEDI domains, and results of the Box and Block Test), median, minimum and maximum values, and interquartile ranges were calculated. For the presentation of dichotomous and ordinal data (sex, GMFCS, and MACS), frequencies and percentages were calculated.

The following PEDI items were selected for analysis: 63, 62, 68, and 71. These items belong to the group of skills related to toileting tasks evaluated in the PEDI.

Fisher's exact test was used to compare the responses of selected items of the PEDI with GMFCS levels. For this analysis, it was necessary to dichotomize the levels of GMFCS: I, II, and III in one group and IV and V in another group. A Wilcoxon test was carried out to compare the BBT results between MACS and GMFCS levels. Additionally, Dunn's multiple comparison test was used.

Spearman's rank correlation was carried out to determine the correlation of the BBT with the PEDI domains. Spearman's rank correlation coefficient in the range 0-0.19 is regarded as very weak, 0.2-0.39 as weak, 0.40-0.59 as moderate, 0.6-0.79 as strong, and 0.8-1 as very strong correlation [24].

Finally, for the selected items related to toileting skills, a Mann-Whitney *U* test was carried out to compare the results of the BBT between children who were able and children who were not able to perform them. They are presented in that order according to the classification of the scale from greater to lesser difficulty.

For statistical analysis, the SPSS software ver. 20.0 (SPSS Inc., Chicago, IL, USA) and GraphPad Prism 7.04 were used. A *p* value < 0.05 was considered statistically significant.

The study was approved by the ethics committee of Universidad Nacional de Colombia by act No. 003-022-17 of February 26, 2017, and the ethics committee of Instituto Roosevelt by act IN-2016-042 of December 16, 2016.

## 3. Results

A total of 162 patients were evaluated. The median age of all patients was 8.9 years (minimum 6.0 and maximum 12.7), and the interquartile range (IQR) was 7.4-10.6. 105 children (64.8%) were male. The distribution of patients by the GMFCS functional level was I = 4 (2,5%), II = 12 (7,4%), III = 36 (22,2%), IV = 97 (59,9%), and V = 13 (8,0%).

The functional characteristics of the patients and the results of the BBT at each of MACS levels are presented in Table 1. The Kruskal-Wallis test showed significant differences in the BBT results between each of MACS levels (*p* ≤ 0.001).

### 3.1. Correlation of the BBT and the PEDI

**Figure 1:**
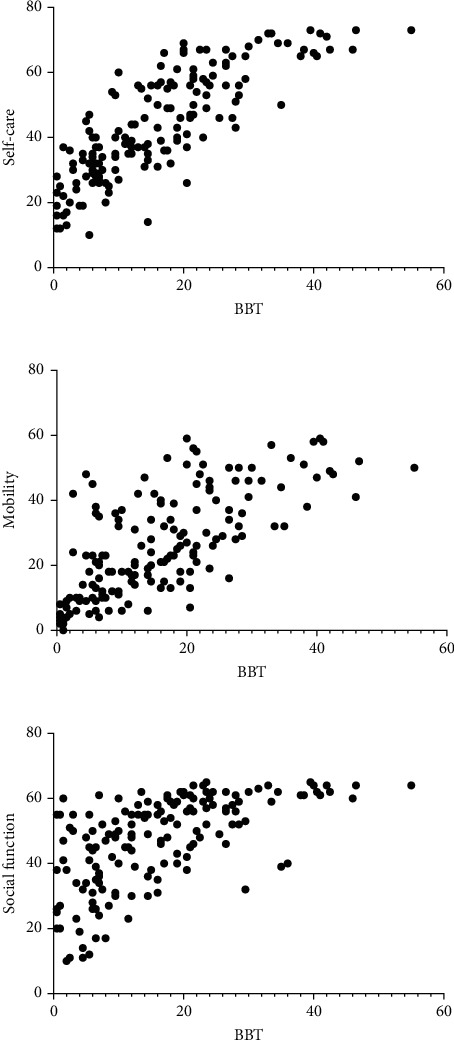
Correlation of the BBT with the PEDI domains.

No correlation of the BBT results with age was found (*r* = 0.13, *p* = 0.08).  Figure 1 shows correlations between the BBT and the PEDI. The BBT showed a very strong correlation with self-care domain (*r* = 0.83; 95% CI = 0.77 − 0.87; *p* ≤ 0.001), a strong correlation with mobility domain (*r* = 0.70; 95% CI = 0.61 − 0.77; *p* ≤ 0.001), and a strong correlation with social function domain (*r* = 0.66; 95% CI = 0.57 − 0.74; *p* ≤ 0.001) of the PEDI (Figure 1).

**Figure 2:**
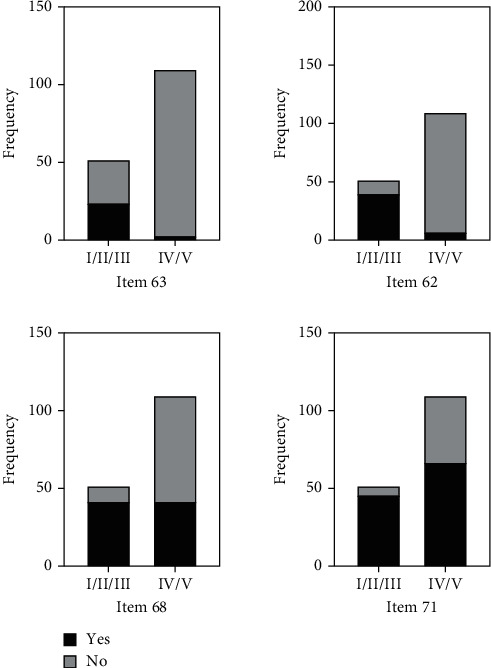
Mastery of selected PEDI items according to GMFCS levels.

Figure 2 shows the PEDI items selected for analysis (63, 62, 68, and 71) with the GMFCS levels.

Items 63 and 62 are related, since they involve manual skills necessary for good functional performance in the bathroom.

In the PEDI scale, item 63 represents one of the most complex and difficult tasks to perform. While 46.1% of the group made up of patients located in levels I, II, and III of the GMFCS managed to perform this item, only 2.7% of the group made up of patients located in levels IV and V managed to perform this item (*p* < 0.0001). Meaning, 3 of 4 children in level I (75%), 8 of 12 children in level II (66.7%), 13 of 36 children in level III (36.1%), 3 of 97 in level IV, and none at level V of the GMFCS managed to perform this item. Item 62 of the PEDI was mastered by 76.9% of the patients located in levels I, II, and III of the GMFCS and by 6.3% of the patients located in levels IV and V (*p* < 0.0001).

Items 68 and 71 are closely related since both are related to sphincter control. Item 68 of the PEDI scale was mastered by 80.7% of the patients located in the group of levels I, II, and III of the GMFCS and by 38.1% of the patients in levels IV and V (*p* < 0.0001). Item 71 of the PEDI scale was mastered by 88.4% of the patients located in the group of levels I, II, and III of the GMFCS and by 60.9% of patients in levels IV and V (*p* < 0.0001).

### 3.2. Correlation of the BBT with the Selected PEDI Items

**Figure 3:**
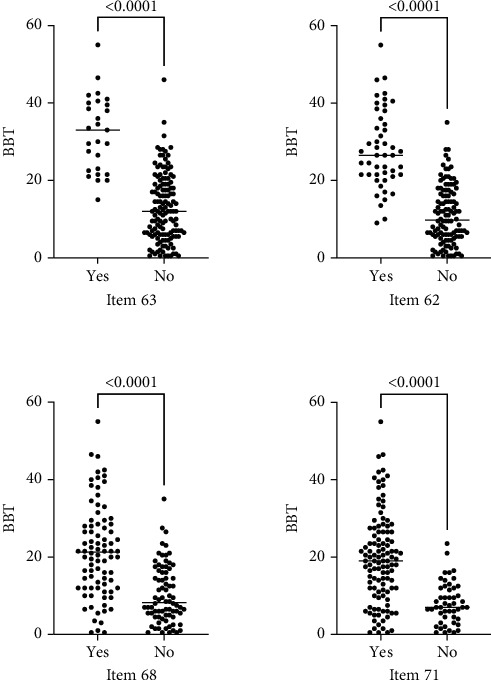
Graphs of the BBT scores and performance of the selected items of toileting (items 63, 62, 68, and 71 of the PEDI scale).

The BBT results and performance of the selected items of toileting (items 63, 62, 68, and 71 of the PEDI scale) showed a significant difference between children capable and incapable of executing those tasks (*p* ≤ 0.001) (Figure 3).

## 4. Discussion

According to the research, the BBT results are correlated with activities of daily living of children with bilateral CP. Significant differences were found between the BBT and MACS levels and a strong or very strong correlation of the BBT with the domains of the PEDI (self-care, mobility, and social function). The data obtained from the test is used to predict the performance of these patients in activities of daily living in settings such as school and home.

A significant lower capacity in the BBT obtained in children with functional status GMFCS III, IV, and V was associated with poor performance in toileting tasks evaluated in the PEDI. Children with the highest BBT scores were able to perform the most complex tasks of the PEDI, for example, item 63, which is the third most difficult task of the functional skills in the self-care domain.

The BBT is easy to apply, and the results are used to describe the functionality of the upper limb of children with CP and to predict possible performance in their daily environment.

The BBT is a normative reference test that assesses fine hand use according to the ICF domain of activity. The PEDI is a norm-referenced test that also can be used as a criterion-referenced test, depending on the purpose of its use and the interpretation of the child's performance in daily life. Criterion-referenced tests consist of task analysis; therefore, they are often more useful for planning intervention programs and measuring changes when used in combination with normative reference tests.

The comprehensive application of capacity and performance assessment measures, such as the BBT and the PEDI, can help establish therapeutic goals in a consensual manner between the rehabilitation team and the family. In addition, evaluation from various perspectives would help to detect atypical cases of children with good capacity and low performance. The combination of different types of tests and questionnaires would make it possible to identify contextual factors that may be affecting the child's functional independence.

In the daily evaluation of children with bilateral CP, the BBT becomes a tool that can be used as a complementary support to establish treatment goals on a realistic basis. For instance, in case some patients have good scores in the BBT, but high dependence on their caregivers in toileting routines is found, the presence of other comorbidities or contextual factors that may be affecting functional independence should be considered.

Toileting tasks evaluated in the PEDI include activities related to being able to manage clothing and wiping after bowel movements. In general, limitations in the activities that children must perform in the bathroom can be a barrier to admission to a regular education school; and this is particularly relevant for children with functional status III, IV, and V. In therapeutic education programs, achieving independence in activities related to toileting skills is a very important goal.

Most children with functional status IV and V are totally dependent on other people for toileting. This limitation extends even to some children with functional status III. Our study showed that bladder and bowel incontinence is a common problem in children with functional status IV and V. 57% of children with functional status IV and all children with functional status V have some degree of incontinence. These findings are similar to other studies [7, 25, 26]. Wright et al. found that at 13 years of age, 50% of children with functional status IV had bladder incontinence day and night [7].

Item 63 (wipes self thoroughly after bowel movements) and item 62 (manages clothes before and after toileting) depend not only on cognitive functions but also on upper limb motor function and gross motor function. Compared with items 71 and 68 (which do not depend on manual skills), scores obtained in the BBT are higher. Item 63 was only mastered by half of the children in the group of levels I, II, and III of the GMFCS. 63.9% of children in level III and 96.9% of children in level IV of the GMFCS failed to master this activity. The BBT scores of the children who mastered this item are the highest, compared to the other selected items of the PEDI. Our study shows that children older than 6 years with bilateral CP, with an average BBT score between 28 and 40, have a high probability of performing item 63 of the PEDI. The correct performance of item 63 requires children to have good balance when sitting and good selective muscle control that allows them to flex the wrist by rotating, extending the shoulder, and extending the elbow.

Item 63 is one of the most difficult tasks in the PEDI scale. More than 90% of healthy children can perform item 63 of the PEDI at 6 years of age [2]. At that age, in healthy children, the results of the BBT have been found to be around 44-49 blocks [15, 27].

Mastery of item 62 involves managing to get clothes up and down; but to get credit for this item, the child should not need help handling clothing. Therefore, it is an item that depends mainly on manual abilities. This explains the high result in the BBT in children capable of mastering this item (median = 26.5). This item gives credit when the clothing is tailored and does not require zippers or buttons. Even so, most of the children in functional status IV and V did not comply with this item. Only 7 of 110 children in GMFCS level IV and none in GMFCS level V managed to perform this item.

Physical access to restrooms and lack of adaptations at home and school may be a factor related to performance failures on items 63 and 62. The benefit of bathroom adaptations has been demonstrated in the functional independence of children with CP [28]. However, adaptations of physical environments do not always exist even in developed countries [5].

Items 68 and 71 are closely related. Item 68 (consistently stays dry, day and night) and item 71 (consistently indicates need to use toilet (bowel)) reflect cognitive functions and maturation of the nervous system. These items do not directly depend on manual ability. This may partly explain why the BBT scores of these two items were lower than items 63 and 62. Bladder and bowel incontinence was a frequent finding in functional levels GMFCS IV and V.

Other studies have shown a strong relationship between manual abilities assessed with the BBT and activities of daily living in patients with CP [1]. The good correlation between the BBT and the MACS scale has also been established (*r* = −0.81, *p* < 0.05) [19]. As in the study mentioned above, in our study, we also used the average of the sum of the right hand and the left hand. However, it is noteworthy that the values obtained in the BBT for levels I, II, and III were lower in our study than those observed by other researchers. While in Ohrvall's study, the average BBT scores for MACS levels I, II, and III were found to be around 40, 30, and 20, respectively, and the averages in our research were 31, 21, and 11 for those same MACS levels. Nevertheless, the populations are not comparable. Our study specifically included children between 6 and 13 years of age with bilateral CP, while Ohrvall's study included patients between 4 and 18 years of age with unilateral and bilateral CP.

Our research has some limitations.

Our study did not evaluate all the psychometric properties of the BBT as that was not the aim of the research. However, it contributes to the construct validity of the BBT in children with bilateral CP. Construct validity is the degree to which the test score is consistent with a hypothesis or theoretical concept based on the assumption that the instrument validly measures the construct to measure [29].

The hypothesis or theoretical concept is that the BBT indirectly represents or is related to the self-care functions of the child with CP. The construct validity is supported by the strong correlation between the BBT and the results of the PEDI, in relation to the self-care of the child with CP. The correlation was very strong with the most related construct (self-care domain of the PEDI (*r* = 0.83)) than for the less related domains of mobility and social function (0.7 and 0.6, respectively).

From the perspective of the COSMIN standards for assessing the quality of studies on reliability and measurement error, the BBT is an example of a performance-based outcome measurement instrument (PerFOMs) in which a professional instructs a patient to comply with a specific manual task [30]. Within this frame of reference, the test–retest reliability of the test has already been reported in healthy children and in children with unilateral and bilateral CP [14, 15]. A comprehensive study of the psychometric properties of the BBT (construct validity, test–retest reliability, minimal clinically important difference, and interpretability) has been carried out in children with unilateral CP [31].

We only included children with bilateral CP and did not determine the test–retest reliability of the BBT. New studies are needed to determine the test–retest reliability of the test in a population with these characteristics.

Among the limitations of the study, it should be mentioned that an evaluation of the intelligence quotient (IQ) was not performed. Cognitive deficit is a factor related to incontinence, manual skills, and overall self-care activities [26, 32] Another important limitation of our study is that most of the patients were in functional groups III, IV, and V.

Finally, the clinical and functional characteristics of the patients evaluated in our research do not necessarily represent the total population between 6 and 13 years old with bilateral CP in our country. Future research should include patients that encompass other regions and distributions to obtain a more representative sample of the population.

## 5. Conclusions

Clinical tests performed in standardized settings correlate with expected performance in the everyday settings of patients with bilateral cerebral palsy. The best test results are obtained by patients with functional levels GMFCS I, II, and III. Based on our findings, patients with functional levels IV and V with poor results in the BBT could be considered to have significant problems in activities of daily living, including toileting, which are associated with significant limitations to be admitted to school.

In a practical setting, in the case of children who show good capacity in the BBT, but who are dependent on hygiene activities in the bathroom, possible contextual factors that are limiting their functional independence should be identified, and therapeutic interventions should be directed to overcome those factors.

This study is a first approach to the functional characteristics of children with bilateral CP between 6 and 13 years old in our community and serves as a basis for future research and treatment proposals.

## Figures and Tables

**Table 1:** General characteristics of patients.

		PEDI			BBT^∗^
		Self-care^∗^	Mobility^∗^	Social function^∗^	
Type	*n* (%)				
Spastic	133 (82.1)	58 (51.3-67.6)	49.7 (36.5-61.4)	62.3 (53.7-70.8)	16 (8.2-23.5)
Dyskinetic	26 (16.0)	51.6 (45.6-57.5)	38.2 (28.1-46.5)	57.5 (45.9-64.3)	6.2 (3.2-16.7)
Ataxic	1 (1.9)	—	—	—	—
Hypotonic	2 (1.2)	—	—	—	—
MACS					
I	9 (5.6)	93 (77.4-96.5)	75.2 (65.5-91.7)	82.2 (72.1-82.2)	41.0 (34.7-46.2)^a^
II	44 (27.2)	67.2 (59.7-75.6)	60.0 (50.7-68.7)	70.8 (59.3-82.2)	22.2 (17.1-28.0)^a,b^
III	62 (38.3)	55.6 (52.4-64.7)	45.2 (40.0-55.0)	60.7 (53.2-66.5)	14.2 (7.3-20.5)^c^
IV	42 (25.9)	47.5 (43.4-53.7)	30.6 (22.7-40.3)	54.3 (46.6-51.1)	6.0 (1.8-11.1)
V	5 (3.1)	40.4 (34.1-46.3)	29.0 (14.8-36.9)	42.5 (37.7-43.4)	3.5 (0.7-5.5)

^∗^Median (IQR). ^a^*p* ≤ 0.001: I vs. III, IV, and V; II vs. IV. ^b^*p* ≤ 0.001: II vs. III. ^c^*p* ≤ 0.001: III vs. IV.

## Data Availability

All data used to support the results of this study are included in the article.
